# Molecular Epidemiology and Antibiotic Resistance Analysis of Non-Typeable *Haemophilus influenzae* (NTHi) in Guangzhou: A Representative City of Southern China

**DOI:** 10.3390/antibiotics12040656

**Published:** 2023-03-28

**Authors:** Shuxian Wen, Ying Mai, Xu Chen, Kun Xiao, Yongping Lin, Zhenbo Xu, Ling Yang

**Affiliations:** 1 Department of Laboratory Medicine, The First Affiliated Hospital of Guangzhou Medical University, Guangzhou 510120, China; 2 Department of Laboratory Medicine, People’s Hospital of HUAIJI, Zhaoqing 526400, China; 3 School of Food Science and Engineering, Guangdong Province Key Laboratory for Green Processing of Natural Products and Product Safety, South China University of Technology, Guangzhou 510640, China; 4 Department of Laboratory Medicine, The Second Affiliated Hospital of Shantou University Medical College, Shantou 515041, China

**Keywords:** non-typeable *Haemophilus influenzae*, antibiotic resistance, multilocus sequence typing

## Abstract

This study aimed to investigate the molecular epidemiology and antibiotic resistance of *Haemophilus influenzae* in Guangzhou, China. A total of 80 *H. influenzae* isolates were collected from the First Affiliated Hospital of Guangzhou Medical University from January 2020 to April 2021. Species identification, antimicrobial susceptibility, molecular capsular typing, multilocus sequence typing and the clinical characteristics analysis of patients were performed. For all recruited isolates, the majority of *H. influenzae* strains from patients with respiratory symptoms were found to be non-typeable *H. influenzae* (NTHi). The isolates were relative susceptible to third- and fourth-generation cephalosporins, quinolones and chloramphenicol, despite having a high ampicillin resistance rate (>70%). The genotyping results reveal a total of 36 sequence types (STs), with ST12 being the most prevalent ST. Remarkably, the 36 STs identified from 80 NTHi isolates within a short period of 15 months and in a single medical setting have revealed a high genetic diversity in NTHi isolates. In comparison, it is noteworthy that the most prevalent STs found in the present study have rarely been found to overlap with those from previous studies. This is the first study on the molecular epidemiology of NTHi isolates in Guangzhou, a city that is representative of southern China.

## 1. Introduction

*Haemophilus influenzae* is a Gram-negative bacterium that initiates infection by colonizing the upper respiratory tract. Acute upper respiratory tract infections in children under the age of five are associated with a high risk of nasopharyngeal carriage of *H. influenzae* [1], and *H. influenzae* is an important microorganism responsible for airway mucosal infections and invasive diseases such as bacterial meningitis. *H. influenzae*, especially *H. influenzae* type b (Hib), is also one of the most common pathogens present in chronic obstructive pulmonary disease (COPD) patients, causing approximately 386,000 deaths annually, 98% of which are patients in underdeveloped countries. Since introduction of the Hib conjugate vaccine, the incidence of infectious disease caused by Hib has decreased over the past ten years. However, infections caused by other *H. influenzae* serotypes and non-typeable *H. influenzae* (NTHi) have grown [2,3].

In some provinces of China, the prevalence of NTHi with high genetic diversity has been reported [4]. Nevertheless, recent data on *H. influenzae*’s molecular epidemiology in southern China are limited. Our previous study found that *H. influenzae* was one of the most common pathogens in children with acute lower respiratory tract infection [5]. Consequently, we aimed to investigate the molecular epidemiology and the antibiotic resistance of *H. influenzae* in Guangzhou, China. Capsular tying and multilocus sequence typing (MLST) were carried out in this study, and we further determined common antibiotics susceptibility and the resistance mechanism of *H. influenzae*.

## 2. Results

### 2.1. The Clinical Information Characteristics of Patients Enrolled

A total of 80 *H. influenzae* isolates were included in this study, with the majority of the isolates coming from children (age 0–18, 32/80, 40%) and elderly patients (age > 60, 32/80, 40%), and from male patients (55/80, 68.75%) twice as often as female (25/80, 31.25%) patients. Isolates from pediatrics, respiratory and other departments accounted for 41.25% (33/80), 38.75% (31/80) and 20% (16/80), respectively. Sputum (54/80, 67.5%) remains the most common specimen type, followed by bronchoalveolar lavage fluid (23/80, 28.75%) and secretion (3/80, 3.75%). Approximately 40% patients had used antibiotics (42.5% vs. 57.5%) or undergone invasive examination (40% vs. 60%) before diagnosis.

### 2.2. Susceptibility of Microbial Antibiotic

The resistance rate of ampicillin was 71.25% in the isolates, and the *β*-lactamase production rate was 63.75%. We had further compared the susceptibility of ampicillin-sensitive and -resistant strains to other antibiotics considering ampicillin is the most common resistant antibiotic for *H. influenzae.* Cefuroxime, azithromycin and clarithromycin resistance levels were significantly higher in ampicillin-resistant isolates than ampicillin-sensitive isolates (*p* < 0.05). In this study, NTHi strains were found to have a relatively low resistance rate to third- and fourth-generation cephalosporins, quinolones and chloramphenicol. The susceptibility of *H. influenzae* isolates to different antibiotics isolates is listed in Table 1.

### 2.3. Molecular Capsule Typing and MLST

In the present study, all the isolates were classified as NTHi using the *bexB*-gene-based PCR method, followed by MLST analysis of 66 *H. influenzae* isolates. According to the results, a total of 35 sequence types were identified among the 66 *H. influenzae* isolates. ST12 (6/66 17.14%) was the most prevalent sequence type, followed by ST1218, ST143, ST107 and ST103 (four strains for each of the four STs). Two strains belonged to ST487 and ST147 each, and only one strain each was found for the other STs. The genetic diversity of strains is shown in Table 2.

### 2.4. Phylogenetic Relatedness and Diversity of Non-Typeable H. influenzae

**Figure 1:**
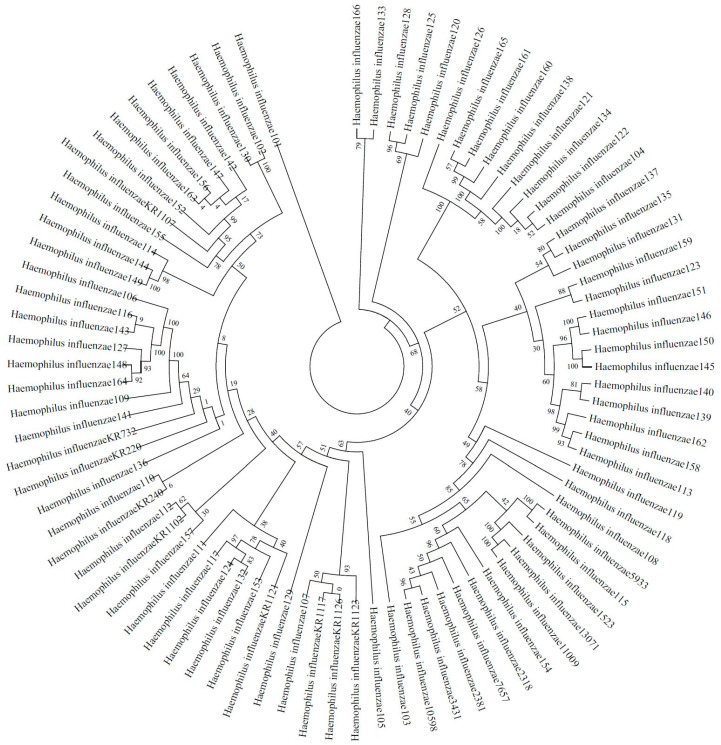
Phylogenetic tree from concatenated sequences of MLST for 66 NTHi isolates isolated from Guangzhou.

A total of 66 *H. influenzae* isolates were included in the phylogenetic diversity analysis, and Figure 1 shows the phylogenetic tree from concatenated sequences of MLST for 66 NTHi isolates. According to the phylogenetic tree, the NTHi isolates can be divided into five columns (strains in the same column or in the neighboring column had a closer relationship than others), exhibiting a highly diverse molecular genetic polymorphism.

## 3. Discussion

According to the molecular capsule typing, *H. influenzae* can be differentiated into encapsulated (typeable) and nonencapsulated (non-typeable) based upon the presence or absence of a capsule. Encapsulated *H. influenzae* (serotype a to f), especially Hib, used to be one of the most common causes of lower respiratory infection until Hib conjugate vaccination became widely used around the world. However, NTHi is widespread and considered to be the primary cause of respiratory tract infection, including acute exacerbations of chronic obstructive pulmonary disease and even invasive diseases [11,12]. In Canada, *H. influenzae* is primarily caused by NTHi, which accounts for 74.2% of cases [12]. Despite the introduction of the Hib vaccine approximately two decades ago, NTHi is still currently widespread in China [4]. Most of the *H. influenzae* clinical isolates were identified as non-typeable *H. influenzae*, and the ratio in some areas of China was surprising high, approaching 100% [4]. In our study, none of the isolates could be typed by the *bexB* gene, from which we inferred that NTHi is a common clinical isolate of acute respiratory tract infection in Guangzhou, which is a representative city of southern China. In the 2000s, Hib was still prevalent among children with pneumonia in China, and even in healthy populations, the colonization rate reached up to 2% at that time. In other developing countries, the colonization rate of Hib may be as high as 15% [13]. According to a recent nationwide investigation on the carriage proportion of *H. influenzae* among healthy populations of China, the overall pooled carriage proportion of *H. influenzae* was 0.17 (95% CI: 0.13–0.21), and the carriage proportion largely varied by province. A higher carriage proportion was found in children than adults and the carriage proportion of NTHi was 22 times than that of Hib [14]. It can be inferred that the carriage proportion of *H. influenzae* is high in chronic obstructive pulmonary disease patients due to the reconstruction of respiratory tract structure, which leads to the recurrence of chronic obstructive pulmonary disease. In the UK, a cross-sectional analysis of sputum samples obtained at the baseline visit in a cohort of stable COPD patients found that approximately two-thirds of the patients had *H. influenzae* carriage in the airways, as measured via qPCR [15]. Microbial colonization has been shown to be detrimental to lung function by affecting the airway epithelium directly and via the recruitment of neutrophils in many pathological conditions [16], such as bronchiectasis and COPD [11,16,17]. Elderly patients, especially chronic obstructive pulmonary disease patients with colonization of pathogens, are considered to have a higher risk of recurrence. After approximately two decades after the introduction of the Hib vaccine in China, the overall carriage proportion of *H. influenzae*, especially Hib, *has* decreased. However, *H. influenzae* is still one of the most common pathogens causing lower respiratory tract infection in children and elderly people. In addition, the change in the prevalent *H. influenzae* type in the present study has raised concern for further prevention and treatment.

In this study, most of the NTHi isolates were isolated from elderly patients and children, which indicates that these two groups are susceptible populations for NTHi infection. In addition, NTHi isolates were more commonly found among male patients, possibly correlating with the higher prevalence of COPD in the male population. In Europe, where the introduction of Hib vaccine took place as early as 1989, NTHi has become the main cause of invasive diseases among persons aged < 1 month and >20, with an increasing annual trend of 3.3% [3]. In addition, *β*-lactamase-negative and *β*-lactam-resistant invasive *H. influenzae* isolates have quickly increased in number in the last decade [18]. In Canada, the number of invasive *H. influenzae* isolates with PBP3 mutations was found to significantly increase during 2007 and 2014 [19]. Multidrug-resistant *H. influenzae* was found in Europe with acquired resistance gene loci and *ftsI* mutations, and *H. influenzae* acquires resistance against a wide range of commonly used antibiotics through horizontal gene transfer, in terms of conjugative transfer of ICEs and transformation of chromosomal genes [20]. Although multidrug resistance is not as serious in Enterobacteriaceae, cefuroxime, azithromycin, and clarithromycin resistance levels are noticeably higher in ampicillin-resistant isolates than ampicillin-sensitive isolates according to our study. Before the Chinese government vigorously promoted the Hib vaccine in 2003, ampicillin-resistant isolates were uncommon, with 88.0% of isolates being susceptible to ampicillin, 100.0% being susceptible to amoxicillin/clavulanic acid, ceftriaxone, cefuroxime and azithromycin, and 99.0% being susceptible to ciprofloxacin according to a prospective muticenter study in China [21]. Most ampicillin resistance was due to beta-lactamase production. ROB-1 and TEM-1 are distinct beta-lactamases that confer high-level resistance on ampicillin and other aminopenicillins [22]. However, *H. influenzae* isolates that are resistant to ampicillin but do not produce beta-lactamase, a phenomenon termed beta-lactamase-negative ampicillin resistance (BLNAR), is becoming increasingly common worldwide. The main mechanism of action of BLNAR is alteration of the PBP3 protein (encoded by the *ftsI* gene). Differences in the level of resistance may be due to different substitutions in the *ftsI* gene. NTHi isolates with rPBP3 variants are classified into three main groups based on the substitution of two key amino acids occurring near the KTG motif, R517H (clustered as group I), N526K (group II) or S385T (group III or III-like), which includes additional substitutions near the SSN motif. The latter confers a higher level of antimicrobial resistance, including resistance to third-generation cephalosporins [23]. However, BLNAR isolates without amino acid substitutions in the PBP3 transpeptidase domain or mutations outside the KTG or SSN motifs, termed miscellaneous mutations, were also found [15]. In Japan, the proportion of low-quinolone-susceptibility isolates has increased significantly [5], and multidrug-resistant *H. influenzae* associated with BLNAR has also emerged [24]. The general resistance rate in NTHi was relatively lower in this study. Similar to our previous study in 2018 [5], resistance in fluoroquinolone was 3.75%, with chloramphenicol, fluoroquinolone and the third-generation cephalosporin remaining sensitive to *H. influenzae*. However, resistance in ampicillin has significantly increased in China compared with two decades before [21].

A number of studies have reported that NTHi has various MLST types, which indicates that phylogenetic diversity can be found among *H. influenzae* strains [8], and whether clustering of *H. influenzae* MLST types is related to antibiotic resistance is controversial. A study had firstly shown a correlation between *ftsI* alleles and MLST types in *H. influenzae* [7]. PBP3 type A is frequently linked to ST14 and ST367 in previous studies [7,25]. A large variety of MLST types in *H. influenzae* has been reported. In Shanghai, a city representative of eastern China, a total of 36 MLST types were identified among 51 clinical isolates [6]. Another study from the USA identified 70 different NTHi MLST types among 165 clinical isolates [8]. In our study, a total of 35 MLST types were found among 66 NTHi isolates within 15 months. As a distinctive feature for this bacterium, high genetic diversity in NTHi isolates has strongly suggested their sporadic emergence and spread in Guangzhou in southern China. ST12 is the most common MLST type, followed by ST1218, ST143, ST107 and ST103. Despite ST12 accounting for up to 17% of strains, there is no evidence showing it is the predominant variety in southern China. In Beijing, a city that is representative of northern China, common MLST types are ST408, ST914, ST57 and ST834 [4], with ST103, ST57 and ST834 being common MLST types in eastern China [6]. A study reported that ST103 was the most prevalent type in the USA [8]. Although ST12 was not the most common MLST sequencing type in China, BLNAR may predominantly distributed in ST12 [26]. However, other major MLST types found in the present study do not belong to prevalent types commonly reported globally, which strongly indicates a high degree of difference in MLST types of NTHi strains from different countries and regions. Nevertheless, its molecular epidemiology and genetical evolution in different countries and regions, as well as the correlation between genotypes and antimicrobial resistance in NTHi, still remain unclear and require further investigation.

## 4. Materials and Methods

### 4.1. Clinical Data Collection

This research was carried out at the first affiliated hospital of Guangzhou Medical University, a comprehensive teaching hospital leading respiratory disease study and treatment in Guangzhou. Patients with *H. influenzae* infection were recruited to our study. Clinical data including sex, age, unit, specimen type, invasive operation and diagnosis were collected. A total of 55 males and 25 females were enrolled. This study was approved by the Ethics Committee at the first affiliated hospital of Guangzhou Medical University. The protocol number of Ethical Committee approval is 2022NO.182.

### 4.2. Isolate Collection and Species Identification

*H. influenzae* isolates were identified in inpatients from January 2020 to April 2021. A total of 80 *H. influenzae* isolates were included in this study (only the first strain isolated from each patient was enrolled). The samples were grown on chocolate agars and incubated for 24 h at 37 °C in air with 5% CO_2_. Suspicious isolates were identified using matrix-assisted laser desorption ionization–time of flight mass spectrometer (bioMérieux, Marcy-l’Étoile, France) and further confirmed via 16S rRNA sequencing. The confirmed colonies (two to three) were homogenized in a reaction tube containing nano-pure water (100 μL) and stored at −80 °C for DNA extraction. DNA extraction was performed via the direct boiling method. The bacterial suspension was thawed at room temperature, followed by vigorous homogenization via vortexing for 30 s. The suspensions were incubated at 100 °C in a boiling water bath for 15 min and immediately frozen at 0 °C on ice. Subsequently, the suspensions were centrifuged at 13,000× *g* at 4 °C for 15 min. The supernatants were transferred to a clean 500 μL tube and stored at −20 °C until PCR analysis [27]. For the 16S rRNA sequencing, DNA was amplified with the following polymerase chain reaction (PCR) procedure: denaturation at 95 °C for 5 min, followed by 30 cycles at 95 °C for 30 s, 55 °C for 30 s and 72 °C for 90 s. Finally, the DNA went through an extended cycle at 72 °C for a further 10 min, and was maintained at 4 °C. The sequences of the PCR products were compared with known 16S rRNA gene sequences in GenBank, as described previously. The primers used for 16S rRNA sequencing were LPW55: AGTTTGATCCTGGCTCAG and LPW56: AGGCCCGGGAACGTATTC AC [28].

### 4.3. Molecular Capsule Typing and Multilocus Sequence Typing (MLST)

Molecular capsule typing was performed via a PCR-based assay using the capsule-producing gene *bexB* in region I and capsule-specific genes (types a to f) in region II of the cap locus, as reported previously [29,30]. The primers used for molecular capsule typing were as follows: *bexB* F:GGTGATTAACGCGTTGCTTATGCG, R:TTGTGCCTGTGCTGGAAGGTTATG. NTHi ATCC49247 and ATCC 9007 were used as the negative and positive control, respectively. For the molecular capsule typing, DNA was amplified with the following polymerase chain reaction (PCR) procedure: 2 min at 95 °C followed by 30 amplification cycles at 95 °C for 30 s, 54 °C for 30 s and 72 °C for 45 s [29].

Seven housekeeping gene fragments, including *adk*, *atpG*, *frdB*, *fuck*, *mdh*, *pgi* and *recA*, were amplified with the following PCR amplification procedure: initial denaturation at 95 °C for 4 min, followed by 30 cycles at 95 °C for 30 s, 55 °C for 30 s and 72 °C for 60 s. The samples were then maintained at 72 °C for 10 min and cooled to 4 °C. Allele numbers and STs were assigned according to the *H. infuenzae* MLST website (http://haemophilus.mlst.net/) [5,30]. Phylogenetic analysis to assess the phylogenetic relatedness and diversity of NTHi was conducted on different isolates with MEGA4 software. The evolutionary history was inferred using the neighbor-joining method. The evolutionary distances were computed using the maximum composite likelihood method and are in units of the number of base substitutions per site [8].

### 4.4. Antimicrobial Susceptibility

Antibiotic susceptibility tests for *H. influenzae* including ampicillin (AMP), ampicillin–sulbactam (AMS), ceftriaxone (CTR), cefuroxime (ROXH), cefotaxime(TAX), meropenem (MEM), trimethoprim–sulfamethoxazole (SXT), azithromycin (AZT), ciprofloxacin (CIP), clarithromycin (CLR) and levofloxacin (LEV) were performed using the Kirby–Bauer disk diffusion method (Oxiod, Basingstoke, Hampshire, England) on *Haemophilus* test medium (HTM) plates, which were then incubated for 24 h at 37 °C in air with 5% CO_2_. Minimal inhibitory concentration (MIC) of AMP was performed using the broth microdilution method. All of the *H. influenzae* isolates were tested for the production of *β*-lactamase using nitrocefin paper disks (bioMérieux, France). Susceptibility results were interpreted according to the 29th Clinical and Laboratory Standards Institute (CLSI) guidelines (Table 2 *Haemophilus influenzae* and *Haemophilus parainfluenzae* inhibition circle diameter and MIC break point, page 74–77).

### 4.5. Statistical Analysis

Data were analyzed with the Statistical Package for Social Sciences for Windows (SPSSWIN, Chicago, IL, USA, version 19.0). Continuous data, shown as mean ± SD or medians, were compared using Student’s t-test or the Mann–Whitney U-test. Categorical variables, summarized as numbers and percentages, were compared using the Chi-square test or Fisher’s exact test. Two-sided *p*-values of less than 0.05 were considered statistically significant.

## 5. Conclusions

In conclusion, NTHi with phylogenetic diversity has become the most prevalent type of *H. influenzae* in Guangzhou, China. ST12 was the most prevalent sequence type in our study. Antibiotic resistance varies by region, but a high ampicillin resistance rate is still the main characteristic of drug resistance. Although muti-drug resistant *H. influenzae* isolates are still uncommon in Guangzhou, the obvious increase in antibiotic resistance among other developed countries in which the Hib vaccine was introduced earlier should grab our attention. It is important to continuously monitor NTHi in the future.

## Figures and Tables

**Table 1:** Antimicrobial susceptibility of NTHi isolates.

Antibiotics	Susceptible vs. Intermediate vs. Resistant	Ampicillin-Resistant NTHi vs. Ampicillin-Sensitive NTHi
Ampicillin	22 (27.5%) vs. 1 (1.25%) vs. 57 (71.25%)	
Ampicillin–sulbactam	45 (56.25%) vs. 0 (0%) vs. 35 (43.75%)	35 (61.4%) vs. 0 (0%), *p* > 0.05
Trimethoprim–sulfamethoxazole	35 (43.75%) vs. 2 (2.5%) vs. 43 (53.75%)	34 (59.65%) vs. 10 (45.45%), *p* > 0.05
Cefuroxime	44 (55%) vs. 6 (7.5%) vs. 30 (37.5%)	30 (52.63%) vs. 2 (9.09%), ***p* < 0.05**
Chloramphenicol	76 (95%) vs. 0 (0%) vs. 4 (5%)	4 (7.02%) vs. 0 (0%), *p* > 0.05
Azithromycin	67 (83.75%) vs. 0 (0%) 13 (16.25%)	29 (50.88%) vs. 1 (4.54%), ***p* < 0.05**
Cefotaxime	77 (96.25%) vs. 0 (0%) vs. 3 (3.75%)	7 (12.28%) vs. 0 (0%), *p* > 0.05
Ciprofloxacin	77 (96.25%) vs. 0 (0%) vs. 3 (3.75%)	5 (8.77%) vs. 0 (0%), *p* > 0.05
Levofloxacin	79 (98.75%) vs. 0 (0%) vs. 1 (1.25%)	3 (5.26%) vs. 0 (0%), *p* > 0.05
Meropenem	77 (96.25%) vs. 0 (0%) vs. 3 (3.75%)	7 (12.28%) vs. 0 (0%), *p* > 0.05
Ceftriaxone	77 (96.25%) vs. 0 (0%) vs. 3 (3.75%)	4 (7.02%) vs. 0 (0%), *p* > 0.05
Clarithromycin	46 (57.5%) vs. 3 (3.75%) vs. 31 (38.75%)	28 (49.12%) vs. 3 (13.63%), ***p* < 0.05**
Moxifloxacin	78 (97.5%) vs. 1 (1.25%) vs. 1 (1.25%)	3 (5.26%) vs. 0 (0%), *p* > 0.05

In the second column, the first number indicates the number of ampicillin-resistant NTHi that are resistant to other antibiotics at the same time, whereas the second number indicates the number of ampicillin-sensitive NTHi that are resistant to other antibiotics. Numbers in brackets indicate the ratio of both antibiotic-resistant NTHi to ampicillin-resistant NTHi and NTHi sensitive to ampicillin but resistant to other antibiotics to ampicillin-sensitive NTHi. Statistically signifcant data are in bold.

**Table 2:** Occurrence and prevalence of NTHi in different countries/regions.

Region	Single or Multicenter	Cases	Population	Source	Duration	Total STs	Prevalent STs	References
Guangzhou	Single center	66	Not limited	Sputum, BALF	January 2020 to April 2021	35	ST12 (17.14%)	This study
							ST1218 (5%)	
							ST143 (5%)	
							ST107 (5%)	
							ST103 (5%)	
Beijing	Multicenter	190	Children	Swab samples	January 2014 to December 2015	108	ST408 (5.8%)	[4]
							ST914 (5.3%)	
							ST57 (4.7%)	
							ST834 (3.2%)	
Shanghai	Single center	51	Adult patients	Not mentioned	July 2015 to June 2018	36	ST103 (7.84%)	[6]
							ST57 (5.88%)	
							ST834 (5.88%)	
Norway	Single center	196	Not mentioned	Eye, ear and respiratory tract samples	January 2007 to February 2007	70	ST367 (14.79%)	[7]
							ST396 (8.16%)	
							ST201 (7.65%)	
							ST159 (6.12%)	
							ST14 (5.61%)	
							ST12 (4.08%)	
							ST395 (4.08%)	
							ST57 (3.06%)	
USA	Multicenter	165	Children	Nasopharynx sample throat swab MEF	June 2006 to December 2009	70	ST103 (11.5%)	[8]
Japan	Multicenter	28	Children	Blood and CSF	2008 to 2015	26	ST3 (7.14%)	[9]
							ST84 (7.14%)	
Italy	Single center	67	Not mentioned	Blood and CSF	January 2009 to December 2011	46	ST103 (8.95%)	[10]
							ST139 (7.46%)	
							ST145 (7.46%)	

BALF: bronchoalveolar lavage fluid, MEF: middle ear fluid, CSF: cerebrospinal fluid.

## Data Availability

Not applicable.

## Abbreviations

*H. influenzae*	*Haemophilus influenzae*
NTHi	non-typeable *Haemophilus influenzae*
ST	sequence type
COPD	chronic obstructive pulmonary disease
MLST	multilocus sequence typing
BLNAR	beta-lactmase-negative ampicillin resistance
CLSI	Clinical and Laboratory Standards Institute
